# Pulmonary Hypertension Following the Use of Trastuzumab Biosimilars

**DOI:** 10.1155/crpu/1076907

**Published:** 2026-05-04

**Authors:** Timi Ojo, Kory Cablay, Neveen Emara, Armin Meyer

**Affiliations:** ^1^ University of South Carolina School of Medicine Greenville, Greenville, South Carolina, USA, sc.edu; ^2^ Department of Internal Medicine and Pediatrics, Prisma Health, Greenville, South Carolina, USA; ^3^ Department of Pulmonology and Critical Care Medicine, Prisma Health, Greenville, South Carolina, USA

## Abstract

**Background:**

HER2‐positive breast cancer comprises 14%–20% of breast cancer cases and was previously linked with aggressive progression. Trastuzumab and its biosimilars have improved survival significantly, but their pulmonary toxicities remain underrecognized. While left ventricular dysfunction is a well‐documented adverse effect, pulmonary hypertension, pulmonary arterial hypertension (PAH), and right heart failure are rarely reported.

**Case Presentation:**

We report the case of a 53‐year‐old woman with Stage IV HER2‐positive invasive ductal carcinoma and well‐controlled HIV who presented with shortness of breath, edema, and weakness. She previously completed five cycles of trastuzumab biosimilars (trastuzumab‐anns or trastuzumab‐dttb), Perjeta (pertuzumab), and Taxotere (docetaxel) and then transitioned to maintenance therapy with just trastuzumab‐anns and pertuzumab for one cycle due to neuropathy. Pretreatment and interim echocardiograms showed preserved left ventricular and right ventricular function. Shortly after her last trastuzumab dose, she was hospitalized with severe anasarca, bilateral pleural effusions, and respiratory failure. Right heart catheterization revealed severe precapillary pulmonary hypertension (mPAP 40 mmHg [normal < 20 mmHg], PAWP 8 mmHg [normal ≤ 15 mmHg]), consistent with WHO Group I PAH. Despite aggressive diuresis and respiratory support, her condition deteriorated, and she elected for comfort‐focused care.

**Discussion:**

Although rare, pulmonary vascular complications such as PAH have been linked to HER2‐targeted therapies. Reports from clinical trials, FAERS data, and national registries have documented cases of trastuzumab‐associated PAH, suggesting a possible vascular mechanism, potentially through ACVRL1 pathway involvement. This case highlights the importance of considering pulmonary hypertension as a potential adverse event in patients on trastuzumab, particularly those with pulmonary metastases.

**Conclusion:**

Clinicians should be aware of pulmonary complications in patients receiving HER2‐targeted therapies, even when left ventricular function is preserved. Early recognition and monitoring of right‐sided pressures in high‐risk patients may improve outcomes. This case adds to emerging evidence on trastuzumab′s pulmonary risks.

## 1. Introduction

HER2‐positive breast cancer accounts for approximately 14%–20% of breast cancer cases and was previously associated with aggressive disease behavior and poor prognosis [1, 2]. The development of HER2‐targeted therapies, particularly trastuzumab and its biosimilars, has significantly improved survival outcomes [3, 4]. While cardiotoxicity, primarily manifesting as left ventricular dysfunction, is a well‐documented risk of taking trastuzumab therapies, pulmonary complications such as pulmonary hypertension, pulmonary arterial hypertension (PAH), and right ventricular (RV) failure remain both rare and underrecognized. We present a case of severe, WHO Group I pulmonary hypertension leading to right‐sided heart failure in a patient with metastatic HER2‐positive breast cancer following trastuzumab biosimilar therapy.

## 2. Case

A 53‐year‐old woman with a history of Stage IV HER2‐positive invasive ductal carcinoma and controlled HIV on consistent antiretroviral therapy presented to the emergency department with progressive shortness of breath and generalized weakness over 4 days. On arrival, she was tachycardic and tachypneic, requiring 4 L of supplemental oxygen. Physical examination revealed significant bilateral lower extremity edema and marked lymphedema of the left upper extremity. Imaging demonstrated bilateral pleural effusions, cardiomegaly, and interstitial opacifications concerning for pulmonary edema.

Eight months prior, the patient was diagnosed with invasive ductal carcinoma after presenting with necrotic autolysis leading to automastectomy. Imaging at that time was notable for metastases to the lung, liver, and bone. Tumor markers included CA 27‐29 at 60.4 U/mL (normal < 38 U/mL), CA 15‐3 at 53.9 U/mL (normal < 30 U/mL), and CEA at 34 ng/mL (normal < 3 ng/mL in nonsmokers). Due to low estrogen receptor expression, hormonal therapy was not pursued. Pretreatment echocardiography showed a left ventricular ejection fraction (LVEF) of 55%–65% (normal 55%–70%) with normal RV function.

She began a palliative regimen of trastuzumab biosimilars, Perjeta (pertuzumab), and Taxotere (docetaxel) every 3 weeks. All the patients′ cycles were completed with trastuzumab‐anns with the exception of Cycle 5, which was completed with trastuzumab‐dttb due to medication availability. After five cycles, and approximately 1 month prior to hospitalization, she transitioned to maintenance trastuzumab‐anns and pertuzumab due to significant neuropathy with dose‐reduced docetaxel. A repeat echocardiogram at that time showed normal LVEF and RV function. Her last infusion prior to hospitalization occurred 15 days before admission, in which she tolerated trastuzumab‐anns and pertuzumab without difficulty. This was her first maintenance infusion after the removal of docetaxel. HIV levels remained undetectable throughout her chemotherapy regimen, and the patient did not miss any doses of her antiretroviral treatments.

**Figure 1:**
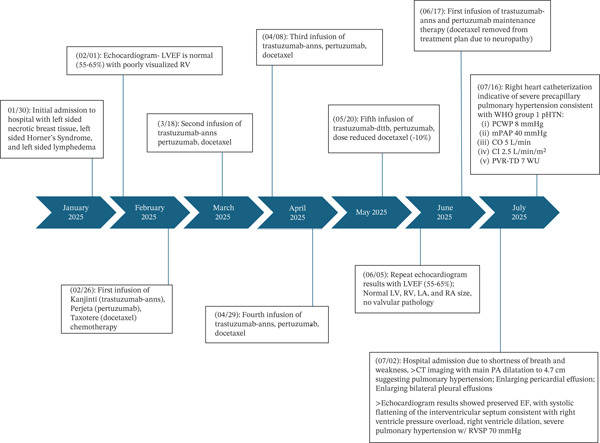
Timeline of interventions and clinical progression.

Once admitted, the patient was placed on noninvasive ventilation and started on continuous furosemide infusion for diuresis. An echocardiogram performed showed preserved LVEF but demonstrated systolic flattening of the interventricular septum, severe pulmonary hypertension with an RVSP of 70 mmHg (normal ≤ 35 mmHg), and RV dilation. She was weaned to a high‐flow nasal cannula after receiving serial thoracenteses for bilateral pleural effusions and multiple days of IV diuresis. However, the patient continued to have pronounced anasarca with intravascular volume depletion. She subsequently underwent right heart catheterization, which was notable for a mean pulmonary artery pressure (mPAP) of 40 mmHg (normal < 20 mmHg), a right atrial pressure of 8 mmHg (normal 2–6 mmHg), and a pulmonary artery wedge pressure (PAWP) of 8 mmHg (normal ≤ 15 mmHg). Cardiac output was 5 L/min (normal 4–8 L/min), and cardiac index was 2.5 L/min/m^2^ (normal 2.5–4.0 L/min/m^2^). Pulmonary vascular resistance via thermal dilution (PVR‐TD) was 7 Wood units (normal < 3 Wood units). As such, the patient was diagnosed with severe, precapillary pulmonary hypertension, WHO Group I, likely due to chemotherapy, given that her HIV was well controlled. Unfortunately, the patient′s respiratory failure continued to worsen, and she elected to transition to comfort‐focused care, where she passed shortly thereafter. A detailed overview of the interventions and clinical progression is provided in the timeline (see Figure 1).

## 3. Discussion

Trastuzumab and its biosimilars have transformed the prognosis of HER2‐positive breast cancer, offering improved survival and quality of life. Cardiotoxicity, especially left ventricular dysfunction, is a well‐documented adverse effect [5]. In contrast, pulmonary complications such as pulmonary hypertension, PAH, and right heart failure are rarely reported, making recognition and diagnosis challenging.

Early‐phase studies of trastuzumab emtansine (T‐DM1) reported isolated cases of Grade 3 pulmonary hypertension, typically in patients with pre‐existing pleural or pulmonary disease [6]. Pharmacovigilance reviews using the FDA Adverse Event Reporting System (FAERS) identified several PAH cases linked to trastuzumab therapy. These analyses proposed a mechanistic overlap with hereditary hemorrhagic telangiectasia (HHT) via ACVRL1 gene dysregulation, a recognized contributor to PAH pathogenesis [7].

Further evidence from registry‐based studies revealed additional incident cases of trastuzumab‐ or biosimilar‐associated PAH [8]. While these reports remain rare, they suggest HER2‐targeted therapies may exert vascular effects, particularly in patients with baseline pulmonary involvement, such as metastases or prior pleural interventions.

Large randomized trials have established the superior safety profile of trastuzumab‐based therapies compared to regimens like lapatinib plus capecitabine, contributing to their widespread use [9]. However, this case illustrates that even well‐tolerated therapies can result in serious complications, particularly in patients with extensive disease burden and compromised physiologic reserve.

This patient′s clinical decline was rapid and fatal, developing acute right heart failure despite preserved baseline LVEF and no known cardiac disease. Her known pulmonary metastases likely contributed to a synergistic effect with trastuzumab‐induced vascular injury, resulting in cardiopulmonary collapse. The rarity of documented right heart failure in this population emphasizes the need for increased clinical competence.

## 4. Conclusion

This case adds to the limited but growing body of literature describing pulmonary complications associated with HER2‐targeted therapies. Clinicians should maintain a high index of suspicion for symptoms such as dyspnea, hypoxia, or peripheral edema in patients receiving trastuzumab, particularly those with pulmonary involvement.

Further pharmacovigilance efforts and prospective data are needed to clarify incidence, risk factors, and mechanisms underlying these complications. Routine right heart monitoring through echocardiography or biomarkers may aid in early detection and improve patient outcomes in high‐risk populations.

## Funding

No funding was received for this manuscript.

## Consent

All the patients allowed personal data processing, and informed consent was obtained from all individual participants included in the study.

## Conflicts of Interest

The authors declare no conflicts of interest.

## Data Availability

Data sharing is not applicable to this article as no datasets were generated or analyzed during the current study.
